# Percutaneous Radiofrequency Ablation Combined With Transarterial Chemoembolization Plus Sorafenib for Large Hepatocellular Carcinoma Invading the Portal Venous System: A Prospective Randomized Study

**DOI:** 10.3389/fonc.2020.578633

**Published:** 2020-10-23

**Authors:** Xiaoyan Ding, Wei Sun, Jinglong Chen, Wei Li, Yanjun Shen, Xiaodi Guo, Ying Teng, Xiaomin Liu, Shasha Sun, Jianying Wei, Wendong Li, Hui Chen, Bozhi Liu

**Affiliations:** ^1^ Department of Cancer Center, Beijing Ditan Hospital, Capital Medical University, Beijing, China; ^2^ School of Biomedical Engineering, Capital Medical University, Beijing, China

**Keywords:** type I/II portal vein tumor thrombus, percutaneous radiofrequency ablation, sorafenib, transarterial chemoembolization, hepatocellular carcinoma

## Abstract

**Background:**

Hepatocellular carcinoma (HCC) with portal vein tumor thrombosis (PVTT) portends a worse prognosis. The objective of this study was to compare the efficacy of percutaneous radiofrequency ablation (RFA) combined with transarterial chemoembolization (TACE) plus sorafenib to that of the most commonly utilized regimen of TACE plus sorafenib in large HCCs with type I/II PVTT.

**Methods:**

An open-label, single-center, prospective, randomized trial of participants with tumors ≥5 cm and type I/II PVTT was performed. Participants with previously untreated HCCs were divided into two groups: RFA + cTACE + sorafenib (study group, n = 40) and cTACE + sorafenib (control group, n = 40). The primary endpoint was the objective response rate (ORR), the secondary endpoints included the overall survival (OS); time to progression (TTP); and toxicity. Prognostic factors were analyzed using cox-regression analysis.

**Results:**

80 patients were enrolled into this study with integrated clinical data. Under a median follow-up of 506 days, the median age was 57.5 years (range: 28–80 years). The ORR of study group was higher than control group (70% vs 22.5%, *p*<0.001). Furthermore, the median OS of study group was superior to that of control group (468 days vs 219 days, HR: 0.44 [95% CI: 0.25–0.78], P = 0.005). Adverse events occurred with 100% probability in both groups (p>0.99), but no treatment-related deaths were recorded. Tumor encapsulation and attaining treatment response predict favorable OS in a multivariate Cox model. The rates of adverse events in both groups were 100% (p>0.99). There were no treatment-related deaths.

**Conclusions:**

RFA combined with TACE plus sorafenib is a safe, well-tolerated three-modality treatment for large HCCs with types I/II PVTT, and it demonstrated better efficacy than TACE plus sorafenib alone.

## Introduction

Hepatocellular carcinoma (HCC), which is categorized as primary liver cancer, is a hotspot in cancer research worldwide due to its high mortality rate (1). Approximately 357,800 new cases of liver cancer were reported in China in 2020 (1). Moreover, 44% to 62% of patients with HCC progress to portal vein invasion, namely portal vein tumor thrombus (PVTT), which has a worse prognosis (2). Previous findings revealed that the median survival time of untreated PVTT patients is only 2.7 to 4.0 months (3). For Barcelona Clinic Liver Cancer (BCLC)-stage C HCC without PVTT, the median overall survival is approximately 1 year (4–6). A uniform tumor thrombus classification system, as described by Cheng, is commonly applied in clinical practice (7). Type I PVTT refers to tumor invading smaller branches of the portal vein in the liver leaf or segment and type II PVTT refers to tumor invading the left and the right branches of the portal vein (7). Although technology was improved thereby promoting the development of HCC therapy, the treatment outcomes of HCC with PVTT remains poor, as expected. Currently, sorafenib or lenvatinib, transarterial chemoembolization (TACE), TACE plus sorafenib, percutaneous radiofrequency ablation (RFA), and radiotherapy, among others, are used in the clinical treatment of PVTT, but these treatment options remain unsatisfactory (8–10). Therefore, further research is urgently needed to investigate the optimal therapeutic strategy for HCC with PVTT.

The use of sorafenib in clinical application enhanced the survival time by 3 months only (5, 6). Besides sorafenib, TACE was always associated with higher overall survival (OS) when compared with the best supportive care in HCC patients with PVTT (9 months vs 6 months) (11). This beneficial effect, however, was only observed in patients with types I–III PVTT (11). Furthermore, a single-center retrospective study involving 557 HCC patients with PVTT found that chemoembolization alone produced a significantly better median time to progression (TTP) and OS (1.6 months and 1.5 months, respectively) than sorafenib treatment alone (12). Notably, sorafenib combined with TACE significantly improved the TTP over sorafenib alone, albeit for no more than 1 month (11–14). The addition of TACE to sorafenib improved a median survival of 5.8 months, in a large cohort of 2112 eligible Child-Pugh A advanced HCC patients with macro-vascular invasion or nodal/distant metastases (10). In a single-center retrospective study involving 57 patients who had a single HCC lesion (≤5 cm) with the main PVTT, RFA of both the HCC and main PVTT significantly prolonged the long-term survival compared to non-treatment (1-year cumulative survival rate: 63% vs 0%, p<0.001) (15). Another retrospective analysis of 134 HCC patients with PVTT whose liver tumor sizes ranged from 2.2 to 16.5 cm revealed a median OS of 29.5 months with TACE combined with RFA (16). However, the usefulness of percutaneous RFA for patients with HCC complicated by PVTT and who have tumors >5 cm remains unconfirmed. The data above indicated that it is clinically necessary to optimize treatment by combining different therapeutic strategies, for instance, combining sorafenib with RFA. Thus, we hypothesize that RFA combined with TACE plus sorafenib will likely result in favorable prognosis in patients with PVTT compared with the regimen of TACE plus sorafenib alone.

The intent of this study was to compare the efficacy of percutaneous radiofrequency ablation (RFA) combined with transarterial chemoembolization (TACE) plus sorafenib to the conventional regimen of TACE plus sorafenib in large HCCs with types I/II PVTT.

## Materials and Methods

### Ethical Approval

This study was conducted in accordance with good clinical practice, guidelines from the Declaration of Helsinki and local organizations, as well as local laws. Documented approval from the institutional review board of Beijing Ditan Hospital, Capital Medical University, was obtained before commencing the study. All participants provided written informed consent before enrollment.

### Study Design

This was an open-label, single-center, prospective, randomized case-control trial. According to the method of random number table *via* version 17.0, SPSS Inc, participants were randomly assigned in a 1:1 ratio to either the RFA combined with TACE plus sorafenib group (study group) or the TACE plus sorafenib group (control group).

### Participants

**Figure 1:**
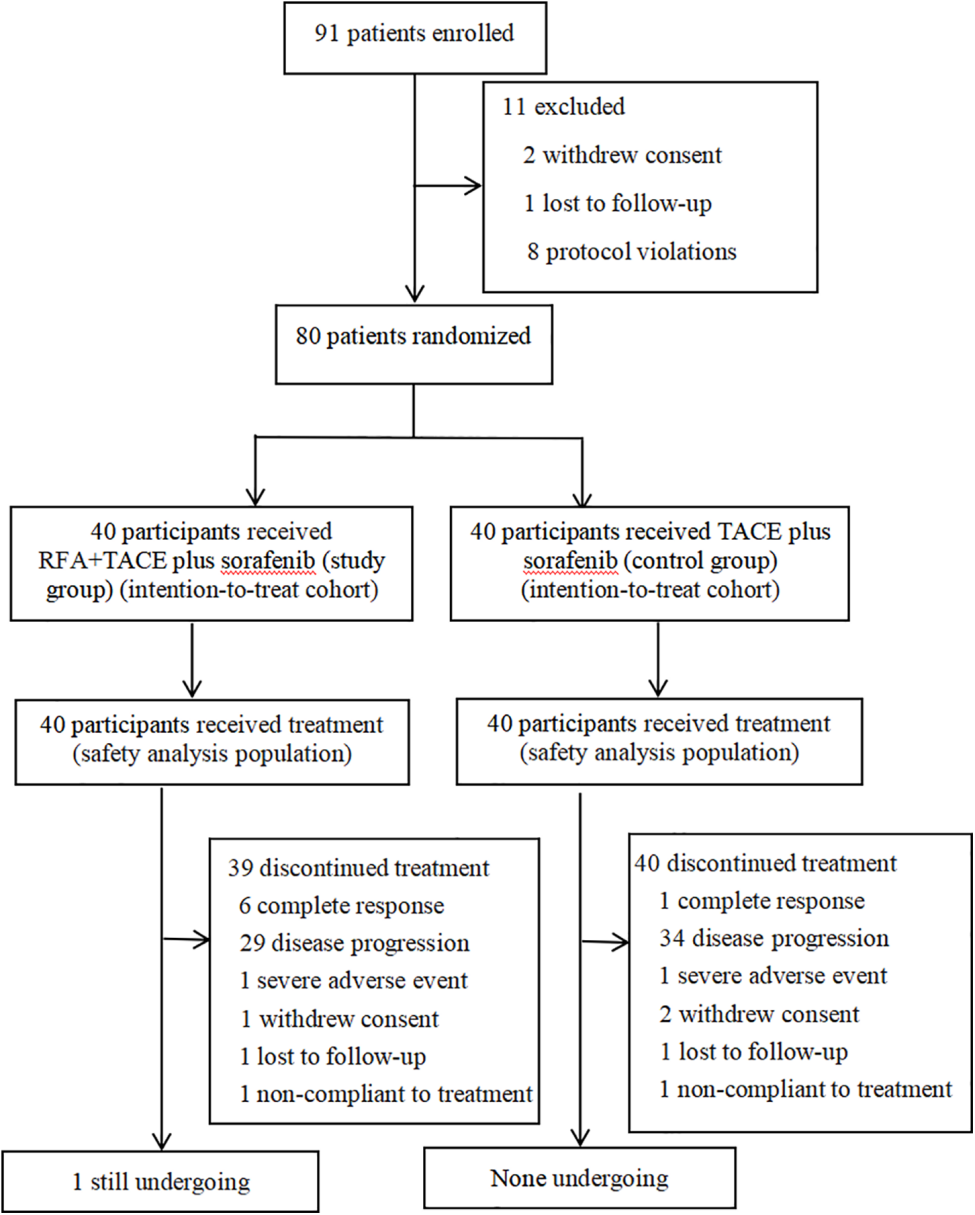
Trial flowchart shows participants selection and treatment diagram. Patients with a complete response discontinued TACE, whereas the other patients discontinued sorafenib. RFA, radiofrequency ablation; TACE, transarterial chemoembolization.

The consecutive participants with HCC complicated by type I/II PVTT who were 18–80 years of age and had not received previous systemic therapy were enrolled in Beijing Ditan Hospital, Capital Medical University between June 2016 and December 2018. During the study period, 91 participants were screened. Of these, two participants withdrew consent, 1 participant was lost to follow-up, and 8 participants had protocol violations (including 3 types III/IV PVTT and 5 with Child-Pugh liver function class C), leaving 80 eligible patients (**Figure 1**).

**Figure 2A:**
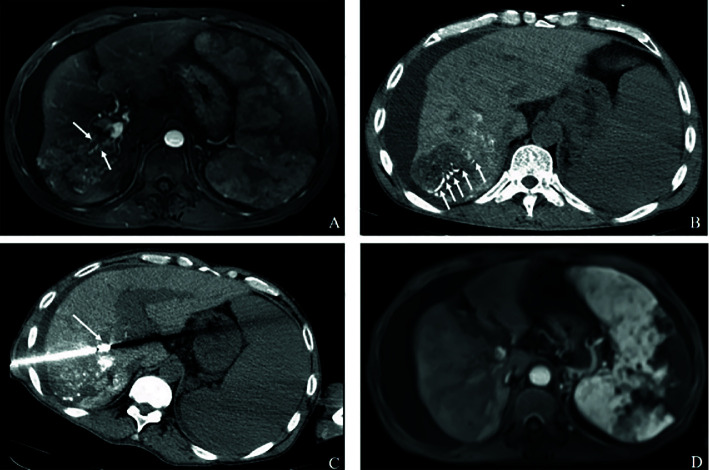
Complete response in a patient with HCC, with right portal vein tumor thrombosis, in RFA+TACE plus sorafenib group. Case 1, male, 72 years old, HCC with right portal vein tumor thrombosis, CR *via* mRECIST criteria, liver tumor relapsed after 120 days until randomization. **(A)** Only one tumor in the right lobe and right portal vein tumor thrombosis (the white arrows). **(B, C)** Treatment with RFA for liver tumor and PVTT, respectively. The white arrows point to the location of radiofrequency ablation electrode. **(D)** No enhancement in both the liver tumor and PVTT.

**Figure 2B:**
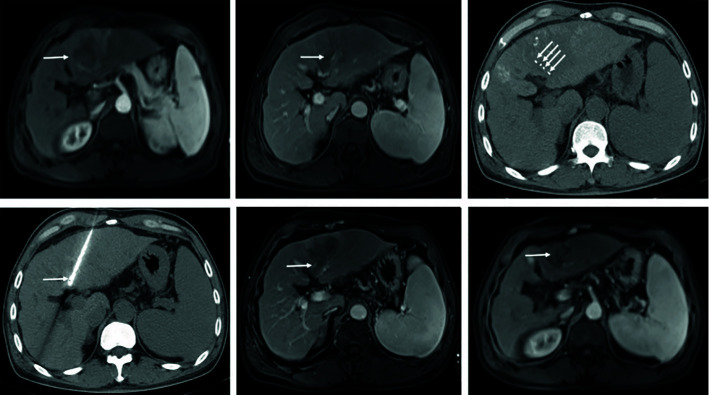
Complete response in a patient with HCC, with left portal vein tumor thrombosis, in RFA+TACE plus sorafenib group. Case 2, male, 50 years old, HCC with left portal vein tumor thrombosis, CR *via* mRECIST criteria, tumor relapsed after 151 days until randomization. **(A)** Liver tumor in the left lobe (the white arrow). **(B)** Left portal vein tumor thrombosis as indicated by the white arrows. **(C, D)** Treatment with RFA for liver tumor and PVTT, respectively. The white arrows point to the location of radiofrequency ablation electrode. **(E, F)** No enhancement in both the liver tumor and PVTT.

**Figure 2C:**
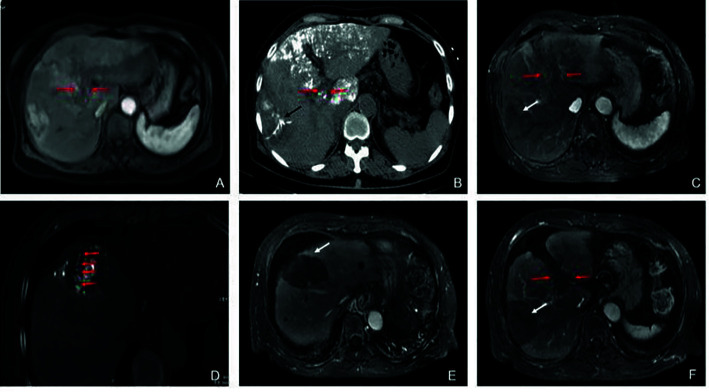
Partial response in a patient with HCC, with left portal vein tumor thrombosis, in RFA+TACE plus sorafenib group. Case 3, male, 65 years old, HCC with left portal vein tumor thrombosis, PR *via* mRECIST criteria, liver tumor progressed after 525 days until randomization. **(A)** 2 liver tumors in the left lobe and the right lobe, respectively, and left PVTT. **(B)** Treatment with the first RFA for liver tumor. The black arrow directs the location of radiofrequency ablation electrode. **(C)** Remnant liver tumor and PVTT. **(D)** Treatment with the second RFA for both remnant liver tumor and PVTT. The red arrow directs the location of radiofrequency ablation electrode. **(E)** With enhancement in the margin of the liver tumor in the left lobe. **(F)** No remnant tumor in the right lobe and the enlarged left PVTT.

**Figure 2D:**
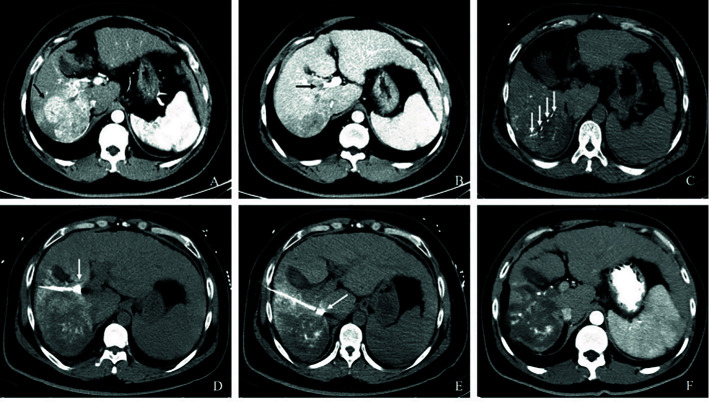
Partial response in a patient with HCC, with right portal vein tumor thrombosis, in RFA+TACE plus sorafenib group. Case 4, male, 58 years old, HCC with right portal vein tumor thrombosis, PR *via* mRECIST criteria, and liver tumor progressed after 158 days until randomization. **(A)** 3 tumors in the right lobe (the black arrow). **(B)** Right portal vein tumor thrombosis shown by the black arrow. **(C)** Treatment with RFA for the largest liver tumor. The white arrow points to the location of radiofrequency ablation electrode (the white arrow). **(D, E)** Treatment with RFA for PVTT. The white arrows point to the location of radiofrequency ablation electrode. **(F)** With enhancement in the margin of the liver tumor in the right lobe and the remnant tumor in the right portal vein thrombosis.

The eligibility criteria were as follows: histologically or cytologically proven HCC; Eastern Cooperative Oncology Group performance status score of 0 or 1; Child-Pugh liver function class A or B ≤8; a life expectancy of at least 3 months; at least 1 tumor lesion >5 cm that had not previously been treated with locoregional therapy and was measurable along a single dimension according to the modified Response Evaluation Criteria in Solid Tumors (mRECIST) (17); and no history of previous locoregional therapy. Participants were required to have adequate renal (i.e. serum creatinine clearance rate of 80 ml/min or more), hematological (i.e. platelet count ≥70×10^9^/L (6), hemoglobin concentration ≥80 g/L, and prothrombin time ≤6 s above control), and hepatic functions (albumin concentration of ≥28 g/L, total bilirubin concentration of ≤51.3 μmol/L, and alanine aminotransferase concentration ≤5 times the upper limit of normal). The exclusion criteria included: type III/IV PVTT; Child-Pugh liver function class C; previous or concomitant systemic therapy (including molecular targeted therapies, herbs, any check-point inhibitors and etc.); known history of HIV infection; clinically serious infections; administered warfarin as an anticoagulant; history of organ allograft; history of cardiac disease; known central nervous system tumor; known gastrointestinal bleeding within 30 days of study enrollment; tumors with 70% or higher liver occupation and pregnancy or breast feeding. All participants were informed of the advantages and disadvantages of the 2 treatment options, including treatment outcomes, treatment-related morbidities, and costs.

### Treatment Protocol

#### Transarterial Chemoembolization (TACE)

TACE was firstly performed by two radiologists (JW and LC, with 6 and 10 years of experience in interventional radiology, separately) as previously described (18). First, the portal vein patency and liver blood supply were confirmed. The participants underwent distal superselective 5-F catheterization of their tumor-feeding hepatic arteries with Embosphere microspheres, lipiodol, and epirubicin. A mixture of epirubicin (50 mg) (Pharmorubicin; Pfizer, Wuxi, China) and lipiodol (5–20 ml) (Lipiodol Ultra-Fluide; André Guerbet Laboratories, AulnaySous-Bois, France) was prepared for TACE. Absorbable Embosphere microspheres (300–500 mm; Biosphere Medical Inc., Rockland, MA) were used for embolization. The entire tumor burden was treated with cTACE. The following cTACE procedures are scheduled at an interval of 8 or 12 weeks for participants with stable disease (SD) or partial response (PR) or progression disease (PD). The subsequent cTACE would be done only if there were no contraindications. The indications were demonstration of viable tumors or intrahepatic recurrences by CT/MRI in patients with favorable clinical and laboratory findings (performance status, liver function, etc.), as well as the absence of vessel casting of both the main portal vein and the branches of portal vein. The number of TACE sessions for both groups was a limit of five.

#### Radiofrequency Ablation (RFA)

RFA was then conducted with a bipolar OLYMPUS electrode (Celon Power, Germany) or multipolar Welfare electrode (WHK-3, Beijing, China) under computed tomography (CT) guidance (SIEMENS, SOMATOM Perspective 64, Germany). RFA was performed by two physicians (W.S. and W. L., with 8 and 10 years of experience in this procedure, separately). Three days ( ± 2 days) after TACE, RFA was performed percutaneously to treat all the reachable intrahepatic tumors in the participants of the study group. Treatment was implemented with local anesthesia and intravenous moderate sedation. Usually, only one liver tumor had been ablated at a time. The adjacent PVTT was also ablated, including the branches of the right portal vein, left portal vein, or both when the portal vein tumor thrombosis caused vessel casting (18). “Vessel casting” means there was no blood flow in the left or/and the right branches of the portal vein and they were filled with tumor. Ablation was often first performed for the intrahepatic tumor, then the portal vein tumor thrombosis was treated. PVTT was ablated according to the strategy reported by Hirooka et al. (19). The electrode ever was placed within the portal vein directly for PVTT which caused “vessel casting” using a bipolar OLYMPUS electrode. But when the PVTT was located in the tumor, multipolar Welfare electrode (WHK-3, Beijing, China) would be used to ablate both the tumor and PVTT simultaneously. The electrode output and ablation time were determined according to technical manuals, and the output for PVTT was set as low as possible (<50 W) to prevent damage to the bile duct or arterial branches. The RFA would be repeatedly performed for the residue liver tumor lesion until active liver burden was not being able to be identified on enhanced CT/MRI (complete ablated, CR) or assessed as PD or RFA-contraindicated. The following RFA could be performed for liver tumors every 8 weeks or 12 weeks. No more than 3 liver lesions would be ablated once a time. The number of RFA sessions for study group was a limit of four. When the participant had any one of the following exclusion criteria, tumors with 70% or higher liver occupation, Child-pugh score was > 8, total bilirubin concentration of >51.3 μmol/L or with types III or IV PVTT, further RFA would be discontinued.

#### Sorafenib

Sorafenib was initially administered wherein the participants were required to take 400 mg sorafenib orally twice daily starting on day 2 after TACE. The treatment was interrupted on the day of TACE or RFA. Moreover, the dose was modified in the event of any severe toxicities. Sorafenib interruptions and dose reductions (first 200 mg twice daily, then 200 mg once daily) were allowed for drug-related toxicity (i.e. intolerable grade 3 hand-foot skin reaction or grade 3 diarrhea and etc.) (6). Sorafenib was administered until unacceptable treatment-related toxicities occurred or when disease progression developed.

### Definition of Endpoints

The primary endpoint was the objective response rate (ORR) as determined *via* mRECIST until the first tumor relapse (17). The best overall response during treatment was considered the final response. The ORR was based on the number of participants who achieved complete response and partial response combined (17). If the participants had no more than five liver lesions, all liver lesions in the cases were defined as target lesions. The PVTT of the cases was considered a non-target lesion. Time to progression (TTP), overall survival (OS) and averse events (AEs) were considered as the secondary endpoints. TTP was measured from the date of randomization until disease progression assessed *via* mRECIST (17). OS was measured from the date of randomization until death from any cause.

### Assessment of Tumor Response and Treatment Safety

Treatment response was evaluated according to mRECIST combined with contrast-enhanced dynamic CT or MRI (17). The target lesions were assessed by two independent radiologists (YX and RX, with 8 and 10 years of experience in radiology, respectively) who were blinded to each other’s findings. Any inconsistencies in their findings were resolved by a third radiologist. In both groups, the tumor response to the entire therapeutic regimen was initially evaluated every 8 weeks. After 6 months, the efficacy was assessed every 12 weeks. Participants whose conditions did not meet the mRECIST definition of complete response (CR), PR, or PD for a minimum of 8 weeks were considered to have stable disease. The ORR was based on the number of participants who achieved complete response and partial response combined (17). Safety was assessed using vital signs, physical examination, clinical and laboratory tests, and AEs every 4 weeks. The AEs were assessed using the National Cancer Institute Common Terminology Criteria for Adverse Events version 4 (20). Information on the safety profile of sorafenib, including rates of sorafenib discontinuation or dose reduction due to AEs, were collected in both groups.

### Statistical Analyses

This prospective parallel trial assumed that the ORR among participants receiving the triplet regimen of TACE combined with RFA plus sorafenib (study group) would be 65%. Among participants receiving only TACE plus sorafenib (control group), the ORR was assumed to be 30%. Both measurements were based on the literature and retrospective data from our center (7, 8). Thus, the superiority margin of the odds ratio for the ORR was deemed to be 1.2, with an α of 0.05 and 1-β of 0.80. As such, 36 participants would be required for each group. However, to account for an assumed dropout rate of 10%, we set a target of 40 participants per group.

Efficacy analysis of all random participants was performed based on the intent-to-treat principle. The safety analysis population consisted of all participants who received at least one dose of sorafenib. The differences in the clinicopathological characteristics between two groups were assessed by Student *t*-test and Chi-square test. Survival curves were estimated using the Kaplan-Meier method and compared with the log-rank test between two groups. A non-parametric log-rank test was used to evaluate the hazard ratios (HRs) and 95% confidence intervals (CIs) of the Cox proportional hazards model. In the univariate analysis, age, sex, family history of primary liver cancer, diabetes, the number of tumors, the treatment, tumor size, extrahepatic metastasis, tumor encapsulation, the type of PVTT, Child-Pugh Class, ALBI, tumor response, and AFP were included to calculate the independent predictors of OS and TTP. Factors that were found to be significant (P<0.10) in the univariate analysis were entered into a multivariable Cox proportional hazards model. All statistical tests were 2-sided, unless otherwise stated *p*<0.05 was considered statistically significant. All statistical analyses were performed using SPSS (version 17.0, SPSS Inc., Chicago, IL).

## Results

### Baseline Characteristics

Finally, 80 were randomly assigned to receive either the triplet regimen of TACE combined with RFA plus sorafenib (study group) or TACE plus sorafenib (control group, n = 40).

The baseline demographics and clinical characteristics of patients are listed in **Table 1**. All 80 participants (median age 57.5 years, range: 28–80 years, 68 men) were among the intention-to-treat population. Most patients in the entire cohort had Eastern Cooperative Oncology Group performance status scores of 1 (n = 64, 80.0%), were Child-Pugh class A (n = 66, 82.5%), and had HBV infection at baseline (n = 71, 88.8%). Most participants had tumors with encapsulation (n = 71, 88.8%), while only 3 participants in control group had extrahepatic metastasis. All of the 80 participants had tumor nodules no more than 5, of whom 18 had only one target liver lesion. The two groups were well-balanced regarding baseline liver function as well as demographic and disease characteristics (**Table 1**).

**Table 1:** Baseline patient demographic and disease characteristics.

	Study group (TACE+RFA-S: n = 40)	Control group (TACE-S: n = 40)	*P* value
Age* (mean ± SD), years			0.684
(57 ± 10 years)	58 ± 10	57 ± 10	
Sex			>0.99
Male (68) Female (12)	34 (85.0) 6 (15.0)	34 (85.0) 6 (15.0)	
ECOG PS score			0.781
0 1	9 (22.5) 31(77.5)	7 (17.5) 33 (82.5)	
Child-Pugh class			0.239
A B	31 (77.5) 9 (22.5)	35 (87.5) 5 (12.5)	
Albumin (g/L)	37.5 ± 4.0	38.0 ± 4.8	0.548
Total bilirubin* (µmol/L)	15.5 ± 0.7	16.5 ± 7.1	0.207
ALBI grade			0.642
1 2	16 (40.0) 24 (60.0)	13(32.5) 27(67.5)	
Hepatitis virus status HBV/HCV/non-HBV or HCV	35(87.5)/2(5)/3(7.5)	36(90)/2(5)/2(5)	0.898
Type 2 diabetes			0.130
Yes No	9 (22.5) 31(67.5)	4 (10.0) 36(90.0)	
Positive family history of primary liver cancer			0.264
Yes No	6 (15.0) 34 (85.0)	2 (5.0) 38 (95.0)	
Type of PVTT			0.479
I/II	6 (15.0)/34 (85.0)	3 (7.5)/37 (92.5)	
Extrahepatic metastasis			0.239
Yes No	0 40(100.0)	3(7.5) 37(97.5)	
Encapsulation			0.479
Yes No	37(92.5) 3(7.5)	34(85.0) 6(15.0)	
Largest diameter*, cm	8.8 ± 2.8	9.8 ± 2.9	0.138
>7.0 cm/≤7.0 cm	27 (67.5)/13 (32.5)	33 (82.5)/7 (17.5)	0.121
Number of liver tumors			
1/2–5 1–3/4–5	11(27.5)/29(62.5) 31 (77.5)/9 (22.5)	7(17.5)/31(82.5) 24(60.0)/16 (40.0)	0.341 0.091
AFP (ng/ml)* ≥100 ng/ml	15803.1 ± 7584 24 (62.5)	5439.4 ± 2380 20 (50.0)	0.264 0.369

Except where indicated, data are numbers of patients, with percentages in parentheses.*t-test, data are means ± standard deviations.

ALBI grade, log10 bilirubin*0.66-albumin*0.085; ALBI, albumin- bilirubin; PVTT, portal vein tumour thrombosis; ECOG PS, Eastern Cooperative Oncology Group performance status; HBV, hepatitis B virus; HCV, hepatitis C virus; AFP, α-fetoprotein; RFA, radiofrequency ablation; TACE, transarterial chemoembolization; S, sorafenib.

### Efficacy Analysis

Until disease progression or death or CR, for the study group the TACE sessions ranged from one to five, and the RFA sessions ranged from one to four. While in the control group, the cTACE sessions ranged from one to three (**Table 2**).

**Table 2:** cTACE sessions and RFA sessions for the entire cohort.

	Study group (TACE+RFA-S: n = 40)	Control group (TACE-S: n = 40)
TACE sessions		
1 2 3 4 5	15 18 6 0 1	27 10 3 0 0
RFA sessions		NA
1 2 3 4	15 18 6 1	

Data are numbers of patients which only included the RFA and/or TACE sessions until disease progression or disease complete response or death.

RFA, radiofrequency ablation; TACE, transarterial chemoembolization; NA, not applicable.

In terms of the best responses, 6 out of 40 participants in study group (15%) achieved a complete response, while 22 (55.0%) had a partial response. In control group, 1 out of 40 participants (2.5%) achieved a complete response while 8 (20.0%) had a partial response. One participant, each in study group and control group, was not evaluable owing to death from a pulmonary embolism and an upper gastrointestinal tract hemorrhage, respectively. The ORR was significantly greater in study group (n = 28, 70.0%) than in control group (n = 9, 22.5%; P<0.001) (**Table 3**). Figures of pretreatment, RFA for liver tumors and PVTT, and post-treatment in 4 cases in the RFA+TACE plus sorafenib group, were depicted in **Figures 2A–D**, which included 2 cases with CR and 2 cases with PR.

**Table 3:** Response rates according to the modified Response Evaluation Criteria in Solid Tumors.

Response	Response rates, n (%)	
	Study group(TACE+RFA-S: n = 40)	Control group(TACE-S: n = 40)
Complete response	6 (15.0)	1 (2.5)
Partial response	22 (55.0)	8 (20.0)
Stable disease	6 (15.0)	16 (40.0)
Progressive disease	5 (12.5)	14 (35.0)

Data are numbers of patients, with percentages in parentheses. RFA, radiofrequency ablation; TACE, transarterial chemoembolization; S, sorafenib; “response” is based on the modified Response Evaluation Criteria for Solid Tumors.

At the end of follow-up, 36 participants in study group and 35 participants in control group had been confirmed with disease progression, respectively. Sixteen participants in study group and 11 participants in control group had chosen Regorafenib as second line systemic drug. Seven participants in study group and 8 participants in control group had chosen mono-lenvatinib as a second line systemic drug. There was no significant difference between various second-line systemic drugs, p = 0.481. Only one participant in control group was treated by lenvatinib plus nivolumab (200-mg ivgtt Q2W) as third-line systemic therapy. The other 13 participants in study group and 16 participants in control group did not take any systemic anti-cancer drugs at all. 17 participants in control group and 28 participants received the following minimally invasive treatment after tumor relapse. No significant difference between with or without minimally invasive treatment after tumor relapse, p = 0.678.

**Figure 3:**
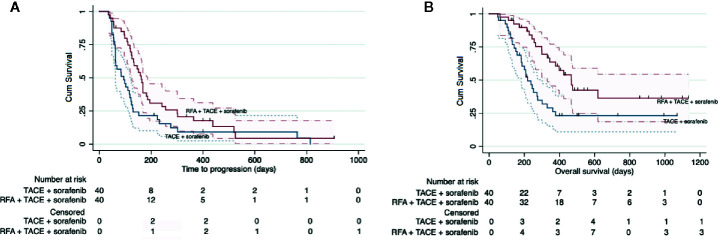
Kaplan-Meier curves showing the time to progression **(A)** and overall survival **(B)** in the whole intent-to treat cohort by the two different treatment regimens: RFA combined with TACE plus sorafenib (study group) and TACE plus sorafenib (control group).

The median follow-up period was 268 days (range, 49–1132 days). The median overall survival was 330 days (95% CI: 233–427 days). Study group had a significantly longer median TTP than control group (162 days [range: 143–181 days] vs 94 days [range, 54–134 days]; HR: 0.58 [95% CI: 0.36–0.93]; P = 0.025; **Figure 3A**). Study group also had a longer median OS than control group (468 days [95% CI: 378–558] vs 219 days [95% CI: 167–271 days]; HR: 0.44 [95% CI: 0.25–0.78]; P = 0.005) (**Figure 3B**).

### Multivariable Analyses

The multivariable Cox proportional hazards model for OS identified tumor encapsulation as a significant positive prognostic factor (HR: 0.39 [95% CI: 0.16–0.99]; P = 0.047). Meanwhile, participants who achieved an objective response had significantly improved prognosis (HR: 0.23 [95% CI: 0.11–0.45]; P<0.001) (**Table 4**). The level of AFP (ng/ml) (p = 0.256), type of PVTT (p = 0.228), and different treatment regimens (p = 0.096) were not associated with improved OS (**Table 3**). The addition of RFA to the therapeutic regimen was not associated with improved TTP (p = 0.72).

**Table 4:** Multivariable Cox proportional hazards model for overall survival.

Variable	HR	*P* Value
Treatment group		
TACE+RFA-S vs TACE-S (reference)	0.67 (0.33, 1.34)	0.256
Type of PVTT		
Type II vs Type I (reference)	2.43 (0.57, 10.27)	0.228
Level of AFP (ng/ml)		
≥400 vs <400 (reference)	1.82 (0.90, 3.68)	0.096
Tumor encapsulation		
Yes vs no (reference)	0.39 (0.16, 0.99)	0.047
Objective response		
Yes vs no (reference)	0.23 (0.11, 0.45)	<0.001

Numbers in parentheses are the 95% confidence interval. An objective response is defined as achieving a complete or partial response based on the modified Response Evaluation Criteria for Solid Tumors.

RFA, radiofrequency ablation; TACE, transarterial chemoembolization; S, sorafenib; HR, hazard ratio; CI, confidence interval; AFP, α-fetoprotein.

### Subgroup Analysis

**Figure 4:**
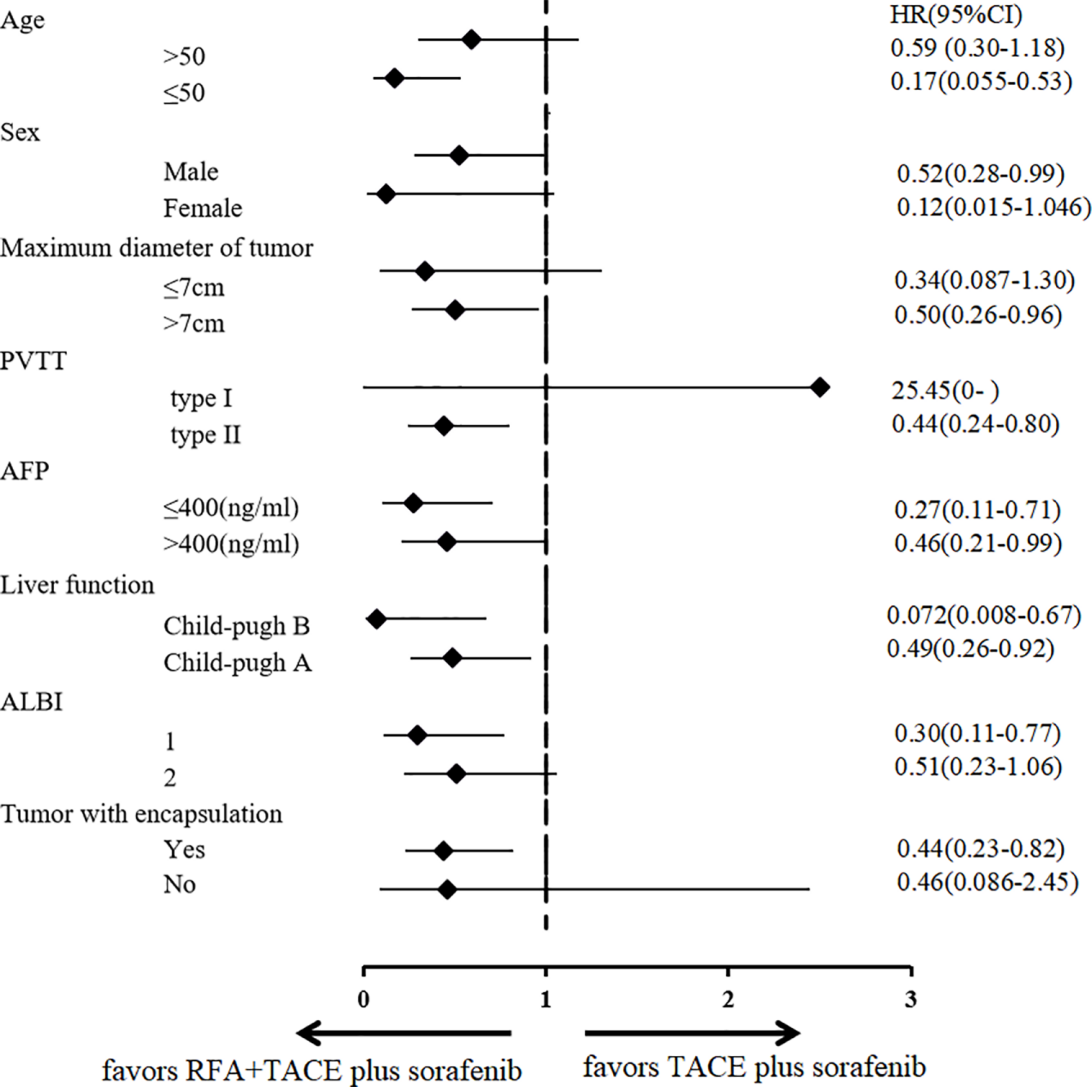
Subgroup analysis according to various prognostic factors. RFA, radiofrequency ablation; TACE, transarterial chemoembolization; AFP, α-fetoprotein; ALBI, albumin-bilirubin grade; PVTT, portal vein tumor thrombosis; HR, hazard ratio; CI, confidence interval.

**Figure 5:**
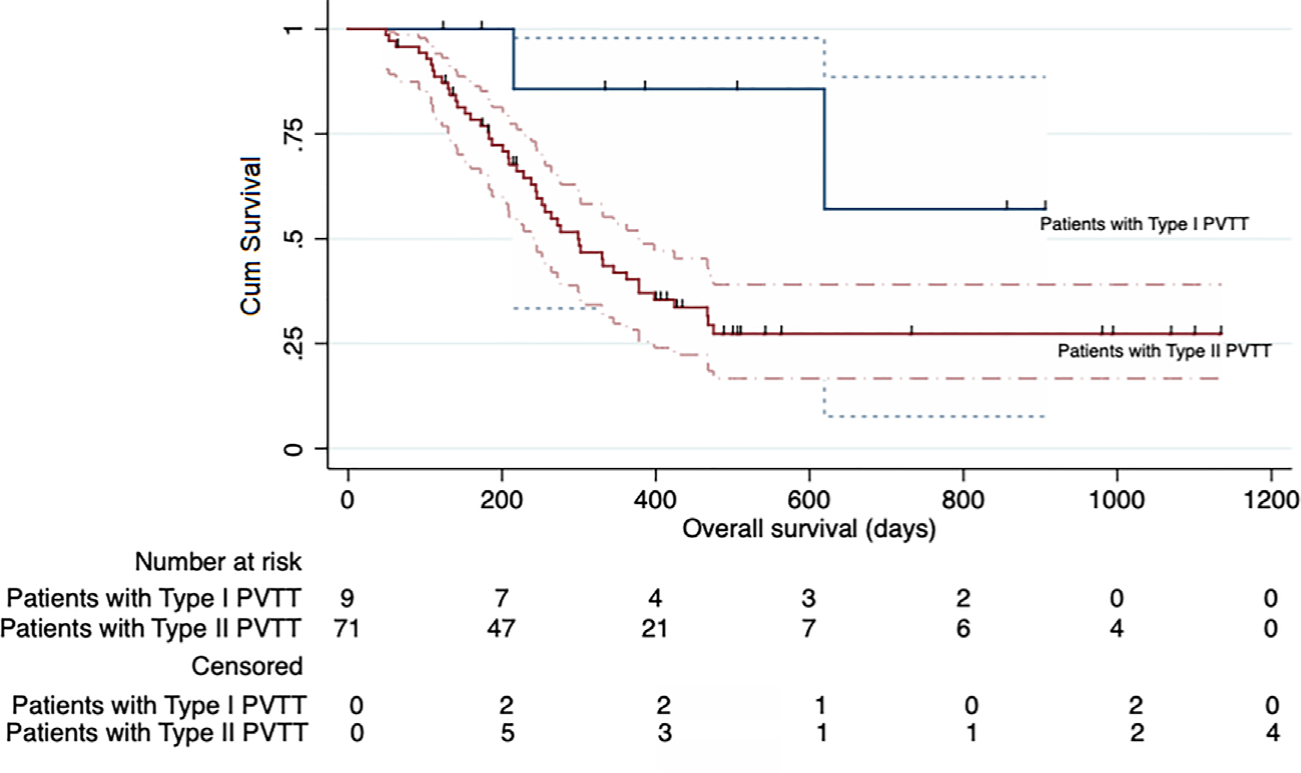
Kaplan-Meier curve showing the overall survival of the participants in the entire cohort according to the type of portal vein tumor thrombosis (PVTT).

TACE combined with RFA plus sorafenib provided clinical benefit in almost all analyzed subgroups, despite some participants having characteristics associated with poor prognosis such as poor liver function (Child-Pugh B and albumin-bilirubin grade 2), a higher AFP level (>400 ng/ml), older age (>50 years), tumor without encapsulation and larger tumor (maximum diameter >7 cm) (**Figure 4**). Although the OS was significantly longer in participants with type I PVTT than it was in those with type II PVTT (median OS: not achieved vs 299 days [95% CI 228–370 days], P = 0.041), the 9 participants with type I PVTT did not benefit from TACE combined with RFA plus sorafenib treatment (p = 0.68) (**Figure 5**).

### Safety

All 80 participants were included in the safety analysis. The overall incidence of treatment-related AEs of any grade was 100% (40 participants) in both study group and control group. Most adverse events were mild to moderate (**Table 5**). The most frequently reported treatment-related AEs (≥10%) were aspartate transaminase elevation (n = 77, 96.2%), alanine transaminase elevation (n = 76, 95.0%), fever (n = 52, 65.0%), anorexia (n = 48, 60.0%), abdominal pain (n = 43, 53.8%), hand-foot skin reaction (n = 38, 47.5%), hypertension (n = 32, 40.0%), fatigue (n = 30, 37.5%), diarrhea (n = 24, 30.0%), weight loss (n = 24, 30.0%), rash or desquamation (n = 20, 25.0%), elevated bilirubin (n = 11, 13.8%), and proteinuria (n = 8, 10.0%). Overall, there were no significant differences in either grade 1/2 or grade 3/4 AEs between the two groups (all p values >0.10) (**Table 4**). One participant in study group developed a liver abscess and was treated with intravenous biapenem and abscess drainage, while another participant in control group developed a liver infection and was treated with intravenous cefotaxime for 10 days; both participants recovered without sequelae. Non treatment-related deaths were reported. Dose reductions and discontinuations were reported in 40.0% (16 of 40) of the participants in study group and 32.5% (13 out of 40) of those in control group (**Table 4**). AEs requiring dose reductions or discontinuation included HFSR, diarrhea, fatigue, and life-threatening upper gastrointestinal hemorrhage.

**Table 5:** Treatment-related adverse events, dose reductions, and discontinuations.

	Study group (TACE+RFA-S: n = 40)		Control group (TACE-S: n = 40)	
	All	Grade 3/4	All	Grade 3/4
Drug-related, n (%)*				
AST elevation	39 (97.5)	11 (27.5)	38 (95.0)	8 (20.0)
ALT elevation	39 (97.5)	10 (25.0)	37 (92.5)	8 (20.0)
Fever	25 (62.5)	5 (12.5)	27 (67.5)	5 (12.5)
Anorexia	25 (62.5)	0	23 (57.5)	0
Abdominal pain	22 (55.0)	5 (12.5)	21 (52.5)	4 (10.0)
HFSR	18 (45.0)	0	20 (50.0)	0
Hypertension	15 (37.5)	0	17 (42.5)	0
Fatigue	15 (37.5)	0	15 (37.5)	0
Diarrhea	14 (35.0)	2(5.0)	10 (25.0)	1 (2.5)
Weight loss	13 (32.5)	0	11 (27.5)	0
Rash/desquamation	9 (22.5)	0	11 (27.5)	0
Bilirubin elevation	6 (15.0)	2(5.0)	5 (12.5)	1 (2.5)
Proteinuria	3 (7.5)	0	5 (12.5)	0
Dose reduction^†^	12 (30.0)		10 (25.0)	
HFSR	7 (17.5)	–	8 (20.0)	–
Diarrhea	3 (7.5)	2 (5.0)	2 (5.0)	1 (2.5)
Fatigue	2 (5.0)	–	0	–
Discontinuation^‡^	4 (10.0)		3 (7.5)	
Hemorrhage, upper GI	2 (5.0)		1 (2.5)	
Diarrhea	–	1 (2.5)	–	1 (2.5)
Grade 3 platelet decrease	–	1 (2.5)	–	–
Fatigue	–	–	1(2.5)	–

Data are numbers of patients, with percentages in parentheses.

*Drug-related adverse events in ≥10% of participants in any study group.

^†^Adverse events causing dose reduction in ≥5% of participants in either study group.

^‡^Adverse events causing discontinuation in ≥2.5% of participants in either study group.

RFA, radiofrequency ablation; TACE, transarterial chemoembolization; S, sorafenib; ALT, alanine transaminase; AST, aspartate transaminase; HFSR, hand-foot skin reaction; GI, gastrointestinal tract.

All of the p values > 0.10.

## Discussion

The median overall survival time for HCCs with PVTT is reported to be only 3 months without any locoregional or systemic drugs or surgery (3). The present guidelines lack consensus on the treatment of unresectable HCC patients with PVTT. Although RFA was not recommended in the American Association for the Study of Liver Disease (21) and European Society for Medical Oncology clinical practice guidelines (22) for HCC with PVTT, two studies showed that TACE plus sorafenib and RFA plus sorafenib are safe and effective regimens (23, 24). Among such patients treated with TACE plus sorafenib, those with PVTT types I-II (Cheng’s classification) showed better outcomes than those with types III-IV PVTT (23). There are two retrospective studies about the three-modality treatment of sorafenib combined with TACE and RFA for treating unresectable HCC (18, 25), but no randomized controlled trials have compared the three-modality treatment to TACE plus sorafenib alone. This means that there is a lack of medical evidence supporting the benefit of the three-modality treatment. Although RFA may benefit HCC with a single liver lesion (≤5 cm) complicated by main PVTT (15), the optimal locoregional treatment regimen for HCC patients with PVTT and liver tumor size >5 cm is still lacking. Our prospective study revealed that, for HCC with types I–II PVTT, liver tumors >5 cm, 88.8% with encapsulation and tumor nodules no more than 5, the three-modality treatment of RFA combined with TACE plus sorafenib resulted in significantly improved OS and ORR without increasing the risk of in-hospital mortality or of AEs. Thus, this new data indicates that for selected HCC patients with PVTT, the three-modality treatment of RFA combined with TACE plus sorafenib might be recommended in clinical practice.

Furthermore, in this study patients with large liver tumors (maximal tumor diameter: >5 cm) were investigated. Our findings showed that sorafenib combined with TACE followed by RFA 3 ± 2 days later is a feasible and effective method in the treatment of large HCCs complicated by types I/II PVTT. Although several studies on this approach have been performed (26, 27), the optimal timing of this combined therapy remains unclear. The majority of clinicians recommend that RFA should be performed 1 week to 1 month after TACE (26); however, in this study, cTACE and RFA were performed sequentially (separated by 3 ± 2 days). An advantage of this near-concurrent treatment with TACE and RFA is the avoidance of lipiodol and chemotherapeutic clearance, which would possibly allow for the formation of new collateral vessels and vascular recanalization (27). TACE effectively inhibited the nutrient vessel supply to the tumor, and it alleviated the effectiveness of blood circulation on heat ablation. Lipiodol has a heat conduction effect; thus, RFA performed after complete lipiodol deposition improves the transduction of heat to peripheral tissues, which increases the ablation effect and reduces the risk of recurrence and metastasis (26, 27). Another advantage of sequential treatment is that tumors with deposited lipiodol can be observed at high contrast in CT. Regions of poor lipiodol deposition were precisely detected by comparing the images obtained after TACE and thus improving the safety of the puncture. Moreover, the presence of necrotic tissue after RFA may induce an immune response against cancer cells (28), with hepatic arterial chemotherapy exerting a synergistic effect with heat ablation (26).

The increased serum levels of vascular endothelial growth factor and vascular endothelial growth factor receptor 2 observed after TACE (29), as well as the incomplete ablation leading to elevated levels of serum hypoxia-inducible factor-1α and vascular endothelial growth factor A (30), indicate that large HCCs with PVTT would require anti-angiogenic drugs such as sorafenib in addition to the combination of TACE and RFA. Our current study showed that participants who underwent the triplet regimen experienced significantly longer OS and TTP than did those treated with TACE plus sorafenib alone (468 vs 219 days and 164 vs 92 days, respectively). Furthermore, TACE combined with RFA plus sorafenib provided a benefit in almost all our participant subgroups, including participants with poor liver function (albumin-bilirubin grade 2), higher AFP value (>400 ng/ml), and larger tumors (maximum diameters >7 cm). The OS was significantly longer in participants with type I PVTT than those with type II PVTT. However, those with type I PVTT did not benefit from the triplet regimen treatment per se. This may be attributed to the fact that there were far fewer participants with type I PVTT in both groups A and B, which would have diluted the statistical power. This may also mean that patients with more advanced disease may benefit more from this triplet regimen of TACE combined with RFA plus sorafenib.

Overall, the treatment-related side effects were mild to moderate, and there were no significant differences in the incidence of grade 1/2 and grade 3/4 AEs between the two groups. One participant in group A developed a liver abscess while another in group B developed liver infection; both participants recovered after intervention. Coincidentally, a previous study by Park et al. found no significant difference in the incidence of liver abscess between their triplet-treated and RFA monotherapy groups of patients with HCC (2.5% vs 2.0%) (31).

Tumor encapsulation was identified as a predictor of favorable OS in our study, which is consistent with previously published data (32–34). Most of the patients had tumors with encapsulation, which may be one of the reasons for such a long median overall survival of 330 days. Collectively, the long survival time and the results of the subgroup analysis suggest that large HCCs with PVTT benefit from the triplet regimen. In contrast from previous literature in patients with HCC who underwent transarterial embolization/chemoembolization-based locoregional treatment with sorafenib or sorafenib monotherapy (32, 33), the AFP level has not been investigated as a marker for predicting treatment response. Although the inclusion of RFA in our study led to significantly improved OS and ORR in participants with large HCCs complicated by PVTT types I/II compared to those receiving only TACE plus sorafenib, RFA administration was not significantly associated with OS on multivariable Cox regression analysis.

Our study had some limitations. Since it was a single-center study, investigator bias and variability in technique with RFA cannot be overlooked. ORR but not TTP or OS, was designated as the primary endpoint. Interval censoring in prospective study has been considered in survival analyses, which may increase the risk of bias in the results of survival time. However, we have strived to establish a multivariate Cox model and subgroup analysis for OS to determine the superiority of the experimental treatment.

In summary, for large HCCs with types I/II PVTT, RFA combined with TACE plus sorafenib demonstrated better efficacy and safety than transarterial chemoembolization plus sorafenib. In the present prospective controlled study, the three-modality treatment can significantly prolong survival time compared to TACE plus sorafenib treatment alone. Additional multicenter prospective randomized controlled trials are needed to validate these findings.

## Data Availability Statement

All datasets presented in this study are included in the article/supplementary material.

## Ethics Statement

The studies involving human participants were reviewed and approved by The Institutional Review Board of Beijing Ditan Hospital, Capital Medical University, Beijing, China. The patients/participants provided their written informed consent to participate in this study. Written informed consent was obtained from the individual(s) for the publication of any potentially identifiable images or data included in this article.

## Author Contributions

JC and WL: conception, design, and funding acquisition. XD and WS: conception, collection and assembly of data, and project administration. JC, WL, XD, WS, YS, XG, YT, SS, XL, JW, WL, HC, and BL: data analysis and interpretation, manuscript writing, and final approval of manuscript. All authors contributed to the article and approved the submitted version.

## Funding

This study was funded by Foundation of Capital Distinctive Clinical Application Research (Project Number: Z161100000516141).

## Conflict of Interest

The authors declare that the research was conducted in the absence of any commercial or financial relationships that could be construed as a potential conflict of interest.
